# Design of Potent and Salt-Insensitive Antimicrobial Branched Peptides

**DOI:** 10.3390/polym15173594

**Published:** 2023-08-29

**Authors:** Janet To, Xiaohong Zhang, James P. Tam

**Affiliations:** Synzymes and Natural Products Center (SYNC), School of Biological Sciences, Nanyang Technological University, 60 Nanyang Drive, Singapore 637551, Singapore

**Keywords:** peptide dendrimers, antimicrobial peptides, polymeric scaffolds, branched peptides, isolysine scaffold, lysine reverse turns

## Abstract

Dendrimeric and branched peptides are polypeptides formed by diverse types of scaffolds to give them different forms. Previously, we reported a cascade-type, Lys-scaffolded antimicrobial peptide dendrimer D4R tethered with four RLYR tetrapeptides. Antimicrobial D4R is broad-spectrum, salt insensitive, and as potent as the natural-occurring tachyplesins, displaying minimum inhibitory concentrations (MIC) < 1 μM. However, the relationships between scaffolds and antimicrobial potency remain undefined. Here, we report the design of four novel types of peptide antimicrobials whose scaffolded backbones are lysine (Lys), iso-Lys, ornithine (Orn), or iso-Orn tethered with RLYR on their α- or sidechain-amines to give ε-, δ-, and their α-branched peptides. When assayed against ten microorganisms, the Lys-scaffolded α- and ε-branched peptides are broadly active, salt insensitive, and as potent as D4R and tachyplesins, whereas the corresponding Orn-scaffolded α- and δ-branched peptides are salt sensitive and much less potent, displaying MICs ranging from 1 to >500 μM. Structure-activity relationship studies suggested that Lys-scaffolds, but not Orn-scaffolds, can support a reverse turn to organize RLYR tetrapeptides as parallel β-strands to form an amphipathic structure with Leu-Tyr as a hydrophobic core. Together, these results provide a structural approach for designing potent and salt-insensitive dendrimeric or branched peptide antimicrobials.

## 1. Introduction

Antimicrobial peptides represent an important innate host defense that often targets microbial membranes, leading to killing mechanisms by increasing membrane permeability, promoting pore formation, or disrupting membrane integrity [1,2,3]. Such membrane-directed antimicrobial killing mechanisms have received increasing attention because of the emerging crisis of antibiotic tolerance and antimicrobial resistance [4]. Indeed, the World Health Organization pronounced that antimicrobial resistance is one of the biggest public health challenges of our time, as thousands of people die from antibiotic-resistant infections each year. Increasing public-health concerns have prompted the need to find effective and affordable solutions to produce peptide antimicrobials.

Antimicrobial peptides are diverse in sequences, forms, and structures [5,6]. In plants, insects, and animals, antimicrobial peptides contain proteinogenic amino acids [1,2,7,8,9]. In contrast, antimicrobial peptides found in bacteria and fungi often contain unusual and nonproteinogenic amino acids, polyamines, and heterocycles in their backbones with cyclic, branched, or other unusual architectures [5,10,11,12,13] that are ribosomally synthesized and post-translationally modified (RiPPs). In addition, polymeric antimicrobials containing γ-, δ-, or ε-peptide backbones are also known [14,15,16,17,18,19]. The antimicrobial ε-isolysines produced by *Streptomyces* and containing 25 to 35 lysine as ε-peptides have been widely used as food preservatives [19,20,21].

**Figure 1:**
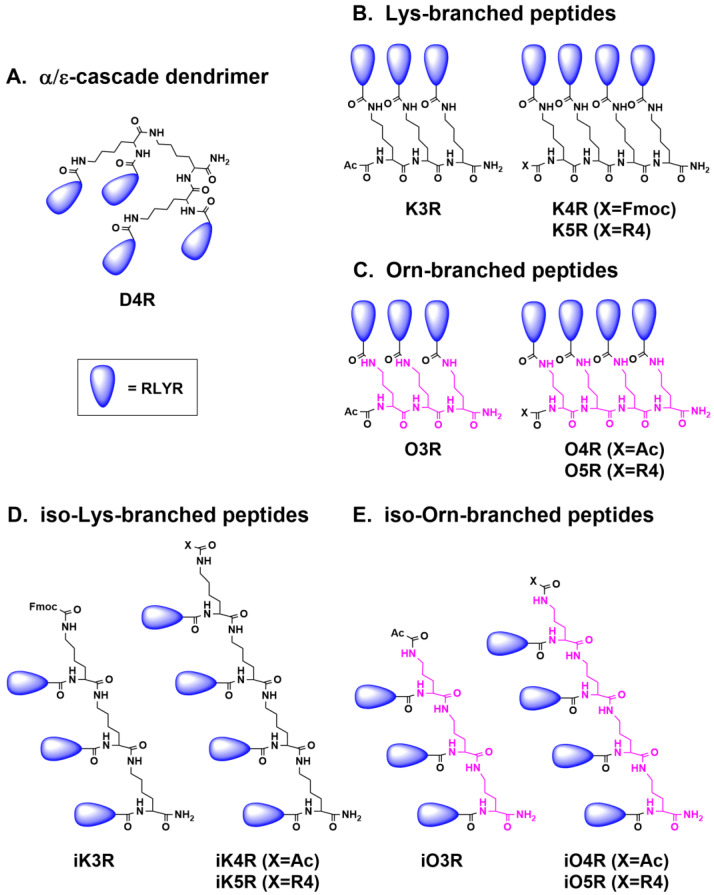
Schematic representation of Lys- and Orn-based scaffolds. (**A**) The Lys-scaffolded α/ε-cascade peptide dendrimer D4R contains a cascade branched tri-Lys scaffold Lys_2_Lys (α/ε-peptides) tethered with 4 strands of RLYR (R4) tetrapeptides, whereas the α-dendrimers (**B**) KnR and (**C**) OnR contain the unbranched, linear tri-, or tetrapeptides of Lys or Orn as the α-peptide scaffolds, tethered with *n* = 3, 4, or 5 strands of RLYR tetrapeptides. (**D**) The ε-branched peptides (iso-Lys) scaffolds include ε-tripeptide in iK3R and ε-tetrapeptides in iK4R and iK5R. Similarly, (**E**) the δ-branched peptides (iso-Orn) scaffolds include δ-tripeptide in iO3R and δ-tetrapeptides in iO4R and iO5R. The unused amino groups of these di-amino scaffolds were employed for tethering the parallel peptide strands. In some cases, the R4 peptides were replaced by a hydrophobic Fmoc protecting group, which is a tricyclic aromatic moiety, or an acetyl protecting group representing a hydrophilic moiety.

Peptide antimicrobials, whether they are dominantly helical or β-strand, share the propensity to be amphipathic by clustering hydrophobic and charge patches that are important for membrane-active functions [22,23]. Indeed, this structural feature has been successfully used to design synthetic cationic β- and γ-peptides, adopting amphipathic helical structures as antimicrobials [24,25]. Previously, we have designed amphipathic β-strand peptide antimicrobials [24,25,26,27]. They include constrained cyclic peptides [26,27,28] based on tachyplesins derived from horseshoe crabs, and protegrins from pig intestines [29,30]. Both antimicrobials belong to cystine-stabilized β-strand peptides consisting of 17 to 19 amino acids. Importantly, they display potent and broad-spectrum antimicrobial activity, often with MICs < 1 μM [29,30]. Interestingly, their antiparallel β-strands can be simplified to a BHHB tetrapeptide motif (B = basic, H = hydrophobic amino acids). To mimic tachyplesins, we have reported a cascade-type antimicrobial peptide dendrimer D4R (Figure 1A) that contains four peptides with a BHHB motif: the tetrapeptide RLYR (called R4 in this paper) tethered at the carboxyl ends of a tri-Lys scaffold [31]. An advantage to producing such peptide antimicrobials is expediency because they can be prepared by controlled polymerization that simplifies their synthesis by many fold, and in turn, lowers their production cost to make them affordable [31,32,33].

D4R is similar in size to tachyplesins and displays similar antimicrobial profiles and potency as tachyplesins even though their forms are different. Tachyplesins and protegrins contain two disulfide bridges that stabilize two β-strands, and which are important for maintaining their antimicrobial activity. Using end-to-end cyclization to replace the disulfide constraints with cyclic tachyplesins and protegrins also retains salt insensitivity [26,27,34]. However, removing the disulfide or cyclic constraints results in a loss of antimicrobial activity under high-salt (physiological) conditions [26,27,29,30]. Thus, it remains to be determined how the unconstrained peptide dendrimer D4R is salt insensitive and maintains its potency like the disulfide-constrained tachyplesins or their cyclic analogs. These findings suggest that the antimicrobial activity of peptide dendrimer D4R may have a structural basis. Furthermore, the salt sensitivity of antimicrobial activity provides a functional correlation in assessing the potency of antimicrobial peptide dendrimers.

Herein, we report the design and structure-activity relationships of four types of linear branched peptide antimicrobials using Orn-, iso-Orn, Lys-, or iso-Lys scaffolds to tether R4 peptides and assess their antimicrobial potency under high-salt conditions (Figure 1B–E). With molecular weights of around 2 kDa, these peptides are dendrimeric polymers of the tetrapeptide R4, which can self-assemble to form high-molecular weight polymers. For comparison, we also prepared the cascade dendrimer D4R. Our results showed that Orn- and Lys-scaffold branched peptides display distinctively different antimicrobial profiles and salt sensitivity. The Lys-based scaffold of iso-Lys ε-branched peptides and their corresponding isomeric α-branched peptides are as broadly active as D4R and tachyplesin-1 when tested against ten organisms under both low- and high-salt conditions. In contrast, the Orn-based scaffold of iso-Orn δ-branched peptides and their corresponding isomeric α-branched peptides are less active and unable to retain their antimicrobial activity against certain bacteria under high-salt conditions. These results provide a structural basis for designing Lys-based scaffolds that could organize parallel R4 peptides into β-structures to affect the antimicrobial activity of dendrimeric and branched peptides under high-salt conditions. 

## 2. Materials and Methods

*Materials*. Solvents, all HPLC grade, Fmoc amino acid derivatives, N-hydroxybenzotriazole (HOBt), N,N-diisopropylethylamine (DIEA), trifluoroacetic acid (TFA), and Fmoc-DPA and Fmoc-MBHA resin (Rink Amide MBHA resin) were obtained from Sigma Aldrich, USA.

Ten organisms obtained from the American Type Culture Collection (ATCC; Rockville, MD) were used for antimicrobial assays. Four Gram-negative bacteria included *Escherichia coli* ATCC 25922, *Pseudomonas aeruginosa* ATCC 27853, *Klebsiella oxytoca* ATCC 49131, and *Proteus vulgaris* ATCC 49132. The three Gram-positive bacteria were *Staphylococcus aureus* 29213, *Micrococcus luteus* ATCC 49,732, and *Enterococcus faecalis* ATCC 29212. The three fungi were *Candida albicans* ATCC 37092, *Candida kefyr* ATCC 37095, and *Candida tropicalis* ATCC 37097. The clinical isolates were obtained from Singapore General Hospital (SGH). They are *Enterococcus faecium*, *Staphylococcus aureus*, *Klebsiella pneumoniae*, *Acinetobacter baumannii*, *Pseudomonas aeruginosa,* and *Enterobacter* species. The strains were incubated in trypticase soy broth (TSB), which was prepared in double distilled water and autoclaved for sterilization. TSB was purchased from Becton-Dickinson (Cockeysville, MD, USA).

*Peptide synthesis and purification*. All peptides were assembled by solid-phase peptide synthesis using Boc- or Fmoc- chemistry [35,36]. Purification and identification were performed by reverse phase-high performance liquid chromatography (HPLC) and MS. Analytical HPLC was conducted on a Shimadzu LG-6A system with a C_18_ Vydac column (4.6 × 250 mm). A linear gradient of 10–90% buffer B ran for 30 min at 1 mL/min with detection at 225 nm. Eluent A: contained 0.04% TFA/H_2_O; B: 0.04% TFA/60% CH_3_CN/H_2_O. Preparative RP-HPLC was performed on a Waters 600 system with a C_18_ Vydac column (20 × 250 mm). Matrix-assisted laser desorption ionization mass spectrometry (MALDI-MS) was measured on a 4800 MALDI TOF/TOF Analyzer (Applied Biosystems, Waltham, MA, USA) instrument. Samples were dissolved in 1 mL of a 1:2 mixture of H_2_O-CH_3_CN. Measurements were made in a linear model, with a-cyano-4-hydroxycinnamic acid (CHCA) as the matrix. 

*Preparation of scaffolds. (a) α/ε-dendrimeric peptide scaffold.* Syntheses of the dendrimer peptide D4R scaffold required two coupling cycles using a four-molar excess of Fmoc-Lys (Fmoc) and BOP/DIEA as a coupling reagent in DMF on the Rink Amide MBHA-resin. The Fmoc group was removed using 20% piperidine/DMF. Each coupling cycle doubled the branching level of the lysyl scaffold to afford the tetrabranching Fmoc-Lys_2_-Lys-MBHA-resin after two cycles. *(b) α-, δ-, and ε-branched peptide scaffolds.* All branched peptide scaffolds were prepared on a TFA-stable diphenylamine (DPA) resin using 3 to 5 equivalents of Fmoc-Lys(Boc) in DMF and BOP/DIEA as the coupling reagent. After removing the Fmoc protecting group, the coupling cycle was repeated two or three times to generate Lys_3_ or Lys_4_ resin supports, respectively, with the Fmoc-protecting group at the N-terminal and the Boc-protecting group at the *α-, δ*-, or ε-amine. Similarly, Orn_3_- and Orn_4_-resin supports were obtained from Fmoc-Orn(Boc), and δ- and ε-peptide resin supports from Boc-Orn(Fmoc) and Boc-Lys(Fmoc) were used as starting materials. 

*Dendrimer Syntheses. (a) α/ε-dendrimer.* Solid-phase peptide synthesis was performed manually using Fmoc-tButyl chemistry and a single coupling protocol with BOP/DIEA in DMF. After peptide chain elongation, the resin was treated with TFA with a scavenge cocktail (TFA:Phenol:Water:EDT:Tris, 86.5:5:5:2.5:1, *v*/*v*) for 4 h. Crude peptides were precipitated in ether and purified by preparative C_18_ reverse-phase HPLC. *(b) α-, δ-, and ε-dendrimers.* R4 peptides were assembled manually by solid-phase synthesis using α-, δ-, or ε-peptide-scaffold resins through conventional Boc chemistry and DCC-HOBt coupling protocol. After completion of the peptide chain assembly, the resin was treated with a high-HF procedure. After the removal of HF, the residual solid was washed with ether. The crude peptides were dissolved in TFA, precipitated in dried ether, and purified by preparative C_18_ reverse-phase HPLC.

*Antimicrobial Assays*. A sensitive and reproducible two-stage radial diffusion antimicrobial assay of Lehrer et al. [37] was employed. A 1–4 × 10^6^ colony-forming unit/mL of the test organism was mixed with 10 mL of molten underlay gel solution and poured into Petri dishes to form a uniform layer. The gel solution consisted of 10 mM sodium phosphate buffer, 0.03% TSB, and 0.02% Tween-20, with the addition of 100 mM NaCl in the case of high-salt conditions. Upon gel solidification, gel wells were made by a 3-mm diameter template in an evenly spaced array. A 5 µL aliquot of a serial half-log dilution of test peptides at seven concentrations was added to each well after removal of gel plugs. The dishes were incubated at 37 °C for 3 h to allow peptide diffusion into the underlay gels. Gels were overlaid with 10 mL of 1% agarose in 6% TSB (wt/vol). After additional incubation at 37 °C for 16–24 h, the diameter of the clear zone surrounding the wells (colony-free) was measured under a microscope. Antimicrobial activities were expressed in units (0.1 mm = 1 unit), and the MICs (in µM) were determined from the x-intercepts of the dose-response curves. Experiments were performed in triplicates.

## 3. Results

### 3.1. Hypothesis and Design

**Figure 2:**
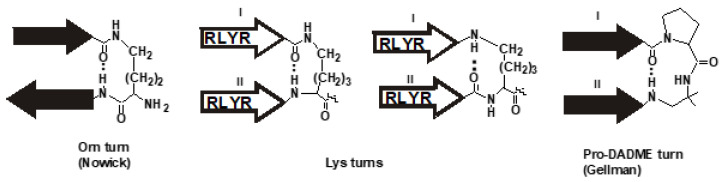
Orn and proposed Lys turns. In an Orn turn, Orn serves as a dipeptide mimetic linking antiparallel strands with an *i*, *i +* 3 10-member hydrogen bond. In the putative Lys turn, the connecting strands are parallel, which could contribute to two Lys turns with a 10-member hydrogen bonding. The D-Pro-DADME (1,2-diamino-1,1-dimethylethane) turn connecting two parallel peptide strands (I and II) has a similar spacing as the Lys turn.

Apart from their disulfide bond constraints, a structural feature found in tachyplesins and protegrins is the reverse turn. In tachyplesin-1, a type-II turn stabilizes the antiparallel β-strands as a β-hairpin [38]. The ability of peptide scaffolds in peptide dendrimers to organize short parallel peptide strands such as R4 to form β-hairpin structures appeared to be worthy of consideration. Gellman and his coworkers [39,40] found that parallel peptides linked by a diamine moiety, D-Pro-DADME (1,2-diamino-1,1-dimethylethane), can fold into stable β-sheet hairpins in water (Figure 2). The Lys linker which forms an ε-peptide in the Lys-scaffold of the cascade branched dendrimer D4R has similar spacing as the D-Pro-DADME linker and could serve as its mimetic. In contrast, Nowick and Brower [41] found that ornithine (Orn) linking anti-parallel peptides can form stable β-hairpin structures through an Orn reverse turn to afford a hairpin structure (Figure 2). However, substituting a Lys-linker for an Orn-linker in the same sequence fails to form a hairpin. Thus, we hypothesized that Orn-scaffold branched peptides could not support reverse turns, but Lys-scaffold branched peptides could form a putative “Lys-turn” to stabilize a β-hairpin structure (Figure 2). 

To test our hypothesis that Lys-scaffolds in α- and ε-branched peptides could form reverse turns to confer structural advantages when compared to an Orn-scaffold in α- and δ-branched peptides, we prepared four series of branched peptides and their analogs containing either a Lys- or Orn-scaffold tethered with R4 peptides (RLYR). The proposed four series of branched peptides organized by either Orn(O)-, isoOrn(iO)- or Lys(K)-, or isoLys(iK)-scaffolds contain four different forms: α-, α′-, δ-, and ε-branched peptides tethered with three to five parallel strands of R4 peptides (*n* = 3 to 5) to give KnR, OnR, iOnR, and iKnR, respectively (Figure 1B–E). Figure 1 also shows their analogs with three to five R4 peptides for structure-activity relationship studies.

### 3.2. Synthesis by Controlled Polymerization

**Figure 3:**
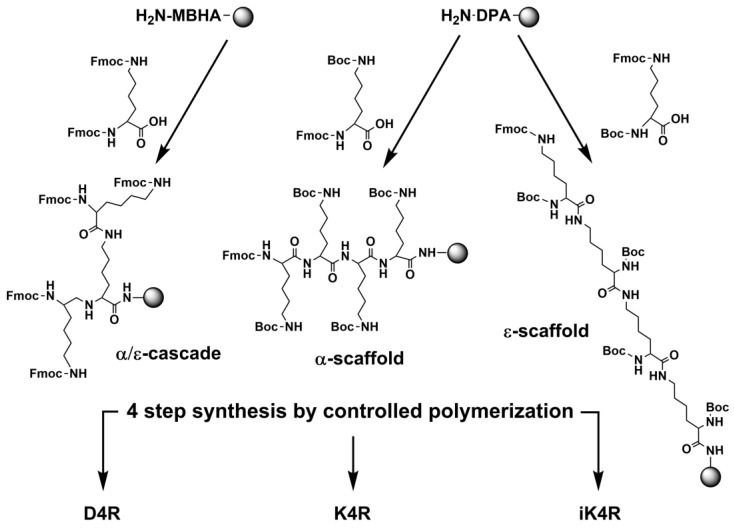
Controlled polymerization schemes of α/ε-cascade dendrimer D4R, and α- and ε-branched peptides K4R and iK4R. The 4-step synthesis of adding the peptide RLYR to these and other dendrimers are common steps in their synthetic schemes. The synthesis of the Orn series was similar to the Lys series, and Boc-Orn(Fmoc)-OH or Fmoc-Orn(Boc)-OH was used.

All branched peptides consisting of 15 to 24 amino acid residues were prepared by a “controlled polymerization” using a stepwise solid-phase method that is procedurally similar to conventional peptide synthesis aside from the stoichiometric adjustments of reagents and protecting group chemistries (Figure 3). In controlled polymerization, multiple peptide strands are assembled simultaneously. Thus, the number of assembling steps in their synthesis was reduced by *n* fold, where *n* is the number of peptide strands. For example, the synthesis of the 17-residue cascade dendrimer D4R with four R4 peptide strands required six assemblage cycles, which is equivalent to the synthesis of a hexapeptide. In contrast, a conventional 17-residue peptide would require 17 synthetic cycles. While the cascade dendrimer D4R was prepared entirely by Fmoc chemistry, the corresponding α-, δ-, and ε-branched peptides [2,7,8,9,10,12,13,42] were prepared by a combination of Boc and Fmoc chemistries. Their α-, δ-, and ε-peptide scaffolds were first assembled by Fmoc chemistry and then R4 peptide strands were tethered using Boc chemistry. All branched peptides were aqueous soluble. They were purified by C18 reversed-phase HPLC and their molecular weights were confirmed by mass-spectrometric analysis.

### 3.3. Antimicrobial Profiles of Orn- and Lys-Scaffold Branched Peptides

The minimum inhibitory concentrations (MICs) of four series of branched peptides and two standards, tachyplesin-1 and the dendrimer D4R, against four Gram-negative bacteria, three Gram-positive bacteria, and three fungi were determined using a two-stage radial diffusion assay in both low- (without NaCl) and high-salt (with 100 mM NaCl) conditions. Under high-salt conditions, tachyplesin-1 was broadly active, displaying MIC values of 0.4 to 1.3 μM against ten test organisms. The cascade dendrimer D4R was also broadly active and salt-insensitive (Table 1). Furthermore, its potency varied within a narrow range, displaying MICs at 0.4 to 1.0 μM under low-salt conditions and 0.6 to 1.9 μM under high-salt conditions. We also tested 31 antibiotic-resistant strains of ESKAPE bacterial pathogens (*Enterococcus faecium*, *Staphylococcus aureus*, *Klebsiella pneumoniae, Acinetobacter baumannii*, *Pseudomonas aeruginosa* and *Enterobacter* species). D4R displayed MICs at 0.3 to 1.6 μM under high-salt conditions (Supplementary Table S1).

**Figure 4:**
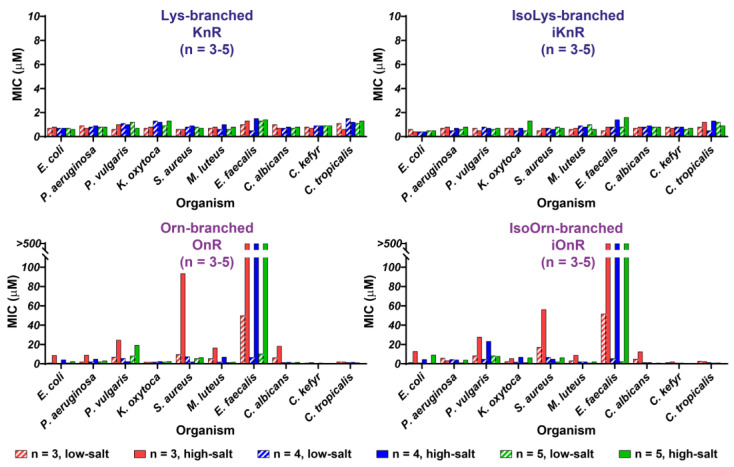
Comparison of antimicrobial activity of Lys-scaffolded α-branched (KnR), iso-Lys ε-branched (iKnR), Orn-scaffolded α′-branched (OnR), and iso-Orn δ-branched (iOnR) peptides, tethered with *n* = 3 (red), 4 (blue), or 5 (green) strands of RLYR tetrapeptides, under low-salt (hatched bars) and high-salt (full bars) conditions. Activities against multiple strains are expressed as the minimum inhibitory concentration (MIC, µM). Experiments were performed in triplicates.

The antimicrobial profiles and potency in the two series of Lys-scaffold α- and ε-branched peptides, irrespective of the number of tethered R4 peptides which range from three to five, are similar and comparable to D4R and tachyplesin-1 (Table 1, Figure 4, and Supplementary Table S2). They exhibited a narrow range of MICs from 0.4 to 1.5 μM under low-salt conditions, and 0.4 to 1.6 μM under high-salt conditions. In contrast, the Orn-scaffold α- and δ-branched peptides exhibited a wide range of MICs from 0.5 to >500 μM (Table 1, Figure 4, and Supplementary Table S2). They were particularly salt-sensitive against certain bacteria. Against *Proteus vulgaris*, *Staphylococcus aureus*, *Micrococcus lutenus*, and *Candida albicans*, the Orn-scaffolded α-peptide dendrimer O3R displayed a 10- to 100-fold decrease in potency under high-salt conditions. 

Unlike the analogous series of Lys-scaffolded α-peptide dendrimer KnR (*n* = 3 to 5 strands of R4), the Orn-scaffolded α-peptide dendrimers OnR (*n* = 3 to 5 strands of R4) improved their potency and salt insensitivity with an increasing number of tethered peptide strands, in the order of O5R > O4R >> O3R. In general, all Orn-scaffold α- or δ-branched peptides were salt sensitive, particularly against the Gram-negative bacteria *Proteus vulgaris*, *Escherichia coli*, and *Klebsiella oxytoca*. They completely lost activity against *Enterococcus faecalis* under high-salt conditions. Interestingly, the salt sensitivity of the Orn-scaffold branched peptides observed in bacteria was less significant in the three test fungi. Also, the increasing number of peptide strands in O3R, O4R, and O5R improved their salt sensitivity against Gram-negative bacteria *Pseudomonas aeruginoa*, *Proteus vulgaris*, and Gram-positive bacteria *Staphylococcus aureus.*

The potencies of the δ-branched peptides improved several fold in certain bacteria when the number of R4 strands in this series increased from three in δ-branched peptide iO3R to four in iO4R. However, there was no further improvement in the δ-branched peptide iO5R with five R4 strands. Despite the increase in potency, their salt sensitivity did not improve significantly with an increase in the number of tethered R4 peptides and the corresponding number of cationic charges. 

### 3.4. Structure-Activity Relationships

**Figure 5:**
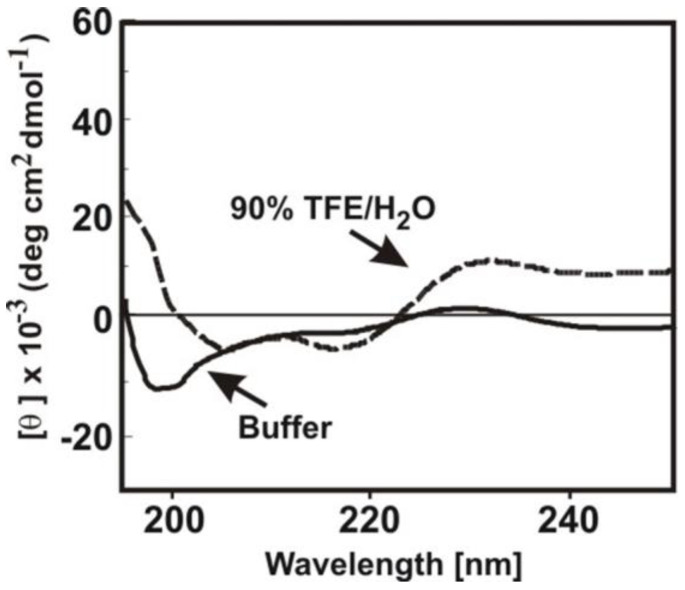
Far-UV circular dichroism (CD) spectra of 10 µM α/ε-Lys-cascade peptide dendrimer D4R in a phosphate buffer with 100 mM NaCl (high-salt) and in 90% TFE/H_2_O.

Because of the symmetry of amino acid sequences in branched or dendrimeric peptides, structure determination by NMR is difficult. Far-UV circular dichroism (CD) of D4R reveals a less intensive spectrum with a negative band at 217 nm, a crossover at 195 nm, and a positive band at about 190 nm (Figure 5), suggesting a β-structure [43,44]. This spectrum is also similar to that of poly(ε-L-Lys), which is proposed to adopt a 40% β-sheet [45]. Using the structure-promoting solvent TFE, the CD of dendrimer D4R reveals a β-turn character [43]. These observations provide partial support for their putative β-structures.

**Figure 6:**
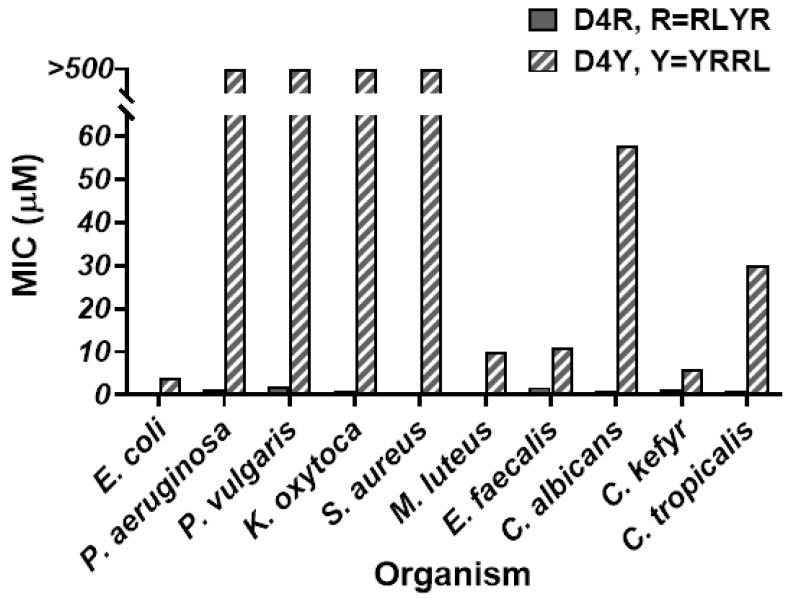
Comparison of antimicrobial activity of D4R (R = RLYR) with a cascade α/ε-Lys scaffold and D4Y (Y4 = YRRL) also with a cascade α/ε-Lys scaffold under high-salt conditions. Experiments were performed in radial diffusion assay with underlay gel containing 1% agarose and a 10 mM phosphate buffer with 100 mM NaCl. Activities against multiple strains are expressed as the minimum inhibitory concentration (MIC, µM). Full bars represent D4R, and hatched bars represent D4Y.

Molecular simulation of D4R with putative Lys turns suggests the importance of a hydrophobic core formed by the Leu-Tyr dipeptide of R4 tetrapeptide. To show that the BHHB motif of the R4 peptide with two consecutive bulky hydrophobic residues in the dendrimeric design is an important contributing factor that facilitates the clustering of hydrophobic and charged regions to form amphipathic structures, we prepared a cascade peptide dendrimer D4Y by replacing the BHHB motif of the R4 (RLYR) peptide with a HBBH (YRRL) motif of the Y4 peptide. Compared to D4R, D4Y was inactive under high-salt conditions against six of ten tested organisms and displayed low potency (MIC < 10 µM) in four other organisms (Figure 6).

## 4. Discussion

In this study, we report that Lys-based scaffolds together with amphipathic structures strongly influence the antimicrobial potency and salt sensitivity of dendrimeric or branched peptides. Lys-based scaffolds tethered with three to five copies of a tetrapeptide RLYR with a BHHB motif can achieve high antimicrobial potency under high-salt physiological conditions. Such antimicrobial activity is retained when tested against the antibiotic-resistant ESKAPE bacterial pathogens, which included the vancomycin-resistant *E. faecium*, methicillin-resistant *S. aureus*, *K. pneumoniae*, *A. baumannii*, and *P. aeruginosa,* and carbapenem-resistant *Enterobacter* species, displaying MICs at about 1 μM or lower under high-salt conditions (Supplementary Table S1). In contrast, similar designs of branched peptides with Orn-based scaffolds are considerably less potent and sensitive to high-salt conditions. We showed that branched peptides are simple to prepare and could provide a cost-efficient approach to developing affordable and potent antimicrobial peptides. 

**Figure 7:**
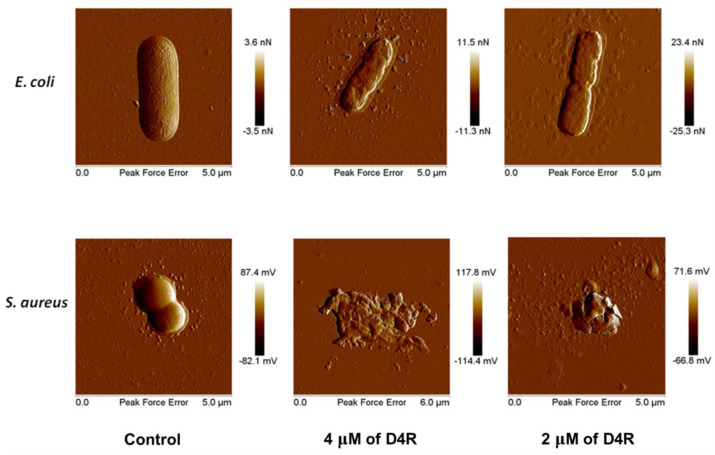
Atomic force microscopy images of the gram-negative bacteria *E. coli* and gram-positive bacteria *S. aureus* treated with 4 μM and 2 μM of the dendrimeric D4R, compared to the untreated controls.

Previously, we compared the antimicrobial profiles and stability of the cascade dendrimer D4R with their corresponding linear polymer (RLYR)_4_; both are similar in size and contain the same number of cationic and hydrophobic residues. D4R and their linear poly-RLYR peptides are nontoxic to red blood cells at their effective antimicrobial IC_50_ of 0.3 to 5 μM [46]. For example, the EC_50_ for hemolysis of human red blood cells for the linear peptide (RLYR)_4_ and D4R are 338 μM and 1510 μM, respectively. Also, consistent with the literature, branched or dendrimeric peptides are more resistant to proteolytic degradation than their linear counterparts. Finally, Figure 7 shows the membranolytic mechanism of D4R against *E. coli* and *S. aureus*, as observed using atomic force microscopy. Together, we can expect the δ- or ε-branched peptides and their respective α-branched peptides reported in this study to share similar attributes as D4R.

From our structure-activity relationship study, we found that the antimicrobial iK3R, an ε-branched peptide with three R4 peptides, displays high potency. Indeed, the 15-residue iK3R represents one of the smallest and most potent antimicrobial peptides we have prepared so far. Increasing the number of peptide strands in the ε-branched peptide series from three to four or five, corresponding to an increase of cationic amino acids from six to eight or ten residues, respectively, did not significantly increase their potency. In contrast, all three forms of α- and δ-branched peptides formed by the Orn-scaffolds are many-fold less potent and are particularly salt-sensitive to certain bacteria, including *Escherichia coli*, *Proteus vulgaris*, *Klebsiella oxytoca*, and *Staphylococcus aureus*. Against *Enterococcus faecalis*, the δ-branched peptides are completely inactive under high-salt conditions. Unlike the ε-branched peptides with Lys-based scaffolds, the δ-branched peptides with Orn-based scaffolds are dependent on the number of R4 peptide strands. Their potency, but not their salt insensitivity, improves with the increase in the number of strands from three to five. Taken together, these results suggest that there is a rough correlation of potency, salt sensitivity, and the number of cationic amino acids with the Orn-based branched peptides, but not the Lys-based antimicrobial branched peptides. The ability of the Orn- and Lys-based scaffolds to shape the structures of these branched peptides may be a major contributor to the observed differences. The Orn scaffolds are unable to form reverse turns to organize parallel peptides as extended structures, and their antimicrobial activity and salt sensitivity improve with an increasing number of R4 strands, and correspondingly, also increase in cationic charges. In contrast, the Lys-scaffolds can form reverse turns to organize parallel peptides to β-structures, and their antimicrobial activity is largely independent of cationic interactions, hence the number of tethered R4 strands. 

A key element in our design is our proposal of a putative “Lys turn” for the Lys-based scaffolds that could stabilize the structures of the ε-peptide branched peptides through a 10-member reverse turn (Figure 2). The proposed putative “Lys turn” differs from the Orn turn in two aspects: the peptide orientation, and the reverse-turn connectivity. The Orn turn is formed by anti-parallel peptide strands that are linked C-terminus-to-N-terminus to the δ-amino and the α-carboxyl groups of the Orn turn, respectively. In contrast, the Lys turn found in the dendrimers or branched peptides is formed by parallel peptide strands that are linked C-terminus-to-C-terminus to the α- and ε-amines of the Lys turn. In the Orn turn, the *i*, *i + 3* hydrogen bond is contributed completed by the anti-parallel peptide strands, and the Orn, a dipeptide mimetic, occupies the *i +* 1 and *i +* 2 positions of the reverse turn. Similar hydrogen bonding for a Lys-turn involving antiparallel strands produces an 11-member ring that is not favored for a reverse turn according to the study by Nowick and Bower [41]. Because of the pseudosymmetry of the parallel strands in a hairpin turn, the *i*, *i +* 3 hydrogen bonding in the “Lys turn” can be contributed by CO of Arg^4^ of the parallel peptide strands and the N^α^H or N^ε^H amine of Lys. Thus, in parallel peptides found in dendrimers or branched peptides, the Orn turn that forms a nine-member β-hairpin turn is not favored. 

**Figure 8:**
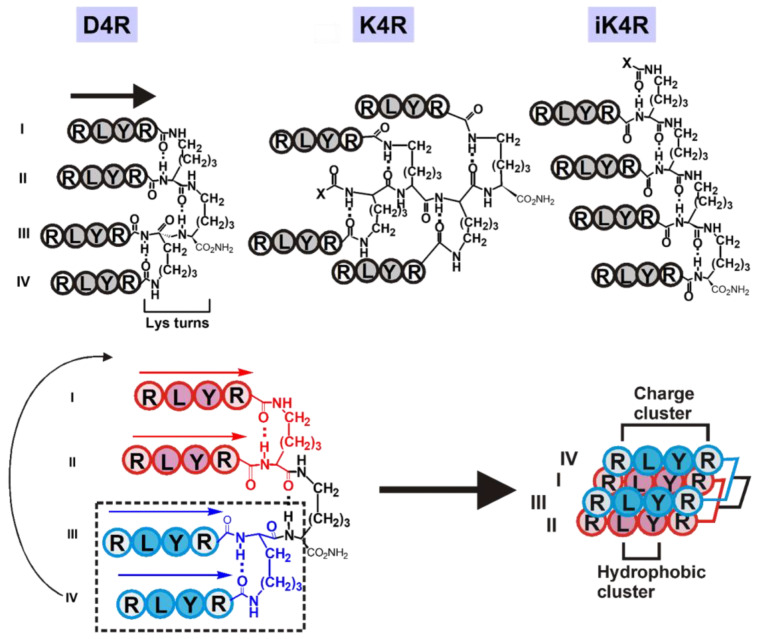
A schematic representation of a proposed amphipathic β-structure for α/ε-Lys-cascade peptide dendrimer D4R, α- and ε-branched peptides K4R and iK4R containing a series of putative Lys turns in their peptide scaffolds. Clustering of hydrophobic residues of the D4R peptide strands would provide an amphipathic β-structure. Roman letters I, II, III, and IV stand for the strand number. The N-to-C orientations of peptide strands are indicated by arrows.

Interestingly, each Lys in the α/ε and ε-scaffolds formed by Lys, either through an α- or ε-peptide linkage, can form a Lys turn. Thus, we can further argue that the trilysine scaffold of α/ε or ε-peptides may form three Lys turns through a series of β-hairpins of intramolecular hydrogen bonding network, most likely through the CO of Arg^4^ to the N^α^H of a Lys-scaffold. These Lys turns in the α/ε- and ε-peptide dendrimers could organize the parallel R4 peptides with a BHHB motif into β-structures such as a β-sheet or β-barrel that cluster the hydrophobic Leu-Tyr (HH residues) as the hydrophobic core and the two Arg at each end to generate bipolar amphipathic structures. Such amphipathic structures would mimic tachyplesins and protegrins, and render the Lys-scaffolded branched peptides and peptide dendrimers salt-insensitive and independent of increased numbers of cationic charges (Figure 8). Since Orn-based scaffolds are not favored to form reverse turns in the Orn-based δ-dendrimers or α- and α/δ-dendrimers, they are unable to form similar β-structures. Their inability to form a stable structure may account for the low antimicrobial activities and salt sensitivity of the δ-dendrimers.

Interestingly, β-strands favor short peptides. Evidence suggested that β-sheets in model peptides become unstable when the strands are lengthened more than seven residues, whereas α-helices become more stable as they grow longer [47]. A plausible explanation is that as the β-strand length grows, the inter-strand interactions that stabilize the β-hairpin are overtaken by the intra-strand interactions of α-helices. This behavior is consistent with analyses of protein crystal structures that show that the prevalence of β-strands decreases steadily as the strands grow longer [48,49]. For example, Eisenberg and his coworkers [50] have extensively examined β-sheets of short peptides to form β-aggregates in amyloid-forming peptides. They have found that many short β-strands of four to six amino acids in length can self-interact to form large β-sheet aggregates. The ability of the putative reverse Lys turns in our Lys-based scaffold may facilitate self-interactions of R4 peptides in the formation of β-structures. It is also consistent with our past observations of α/ε-branched cascade peptide dendrimers, such as the multiple antigen peptides [31,32,33], which generally exhibit α-helical or random structures because the parallel peptide strands attached to the ε-peptide scaffolds as antigens are >10 amino acid residues in length. To minimize immunogenicity, we have used short peptides using oligolysine-based scaffolds.

In conclusion, peptide dendrimers and branched peptides described in this report represent a simple approach to designing and preparing antimicrobial peptides. They are small, consisting of 15 to 24 amino acid residues, and are prepared using a controlled polymerization strategy that is many fold faster than the synthesis of conventional peptides of similar sizes. The Lys-scaffolded dendrimers could support a novel “Lys-turn” to organize the tethered parallel peptides into β-structures that cluster charge and hydrophobic residues to confer the desired potency and salt-insensitivity for antimicrobial peptides.

## Figures and Tables

**Table 1:** Antimicrobial activity of tachyplesin, α/ε-Lys-cascade peptide dendrimer D4R, Lys α-branched (K4R), iso-Lys ε-branched (iK4R), Orn α’-branched (O4R) and iso-Orn δ-branched (iO4R) peptides, tethered with four strands of RLYR tetrapeptides, under low (L-) and high (H-) salt conditions. Activities against multiple strains are expressed as the minimum inhibitory concentration (MIC, µM). Experiments were performed in triplicates.

	MIC (µM) *											
	Controls				Lys-Scaffolded ^†^				Orn-Scaffolded ^†^			
	Tachyplesin		D4R		K4R		iK4R		O4R		iO4R	
Organism	L-Salt	H-Salt	L-Salt	H-Salt	L-Salt	H-Salt	L-Salt	H-Salt	L-Salt	H-Salt	L-Salt	H-Salt
**Gram-negative**												
*E. coli*	0.3	0.4	0.6	0.7	0.7	0.7	0.4	0.4	0.7	**4.1**	0.9	**4.4**
*P. aeruginosa*	0.9	0.5	0.5	1.2	0.8	0.9	0.5	0.7	2.1	**4.8**	**4.4**	**4**
*P. vulgaris*	0.7	1	1	1.9	1.1	1	0.8	0.7	**5.6**	2.2	**4.7**	**23.2**
*K. oxytoca*	0.2	0.5	0.4	0.9	1.3	1.2	0.5	0.7	1.8	2.4	1.6	**6.8**
**Gram-positive**												
*S. aureus*	0.4	0.5	0.8	0.6	0.8	0.9	0.7	0.6	**7.3**	2	**6.7**	**4.8**
*M. luteus*	1	1.1	0.5	0.7	0.6	1	0.9	0.8	1.8	**6.9**	2.1	2
*E. faecalis*	0.3	0.4	0.8	1.8	0.5	1.5	0.8	1.4	**6.6**	**>500**	**5.4**	**>500**
**Fungi**												
*C. albicans*	0.7	0.9	0.8	0.8	0.7	0.8	0.8	0.9	1.4	1.6	1.3	1.4
*C. kefyr*	0.9	1.3	0.9	1.3	0.9	0.9	0.8	0.8	0.7	0.8	0.8	0.8
*C. tropicalis*	0.5	1	0.7	0.8	1.5	1.2	0.5	1.3	1.4	1.5	1.4	0.8

* When MIC is >3 µM, the number is shown in bold. **^†^** See Figure 1 for structures.

## Data Availability

The data presented in this study are available on request from the corresponding author.

## Supplementary Materials

The following supporting information can be downloaded at: https://www.mdpi.com/article/10.3390/polym15173594/s1, Table S1: Antimicrobial activity of dendrimeric D4R against ESKAPE bacterial pathogens under high-salt conditions; Table S2: Antimicrobial activity of dendrimeric and branched peptides with Orn- and Lys-based scaffolds under low- and high-salt conditions.

## Abbreviations

BOP, benzotrizol-1-yl-oxy-tris(dimethylamino)phosphonium-hexafluorophosphate; CHCA, a-cyano-4-hydroxycinnamic acid; D-Pro-DADME, 1,2-diamino-1,1-dimethylethane; DCC, N,N-dicyclohexylcarbodiimide; DCM, dichloromethane; DIC, N,N-diisopropylcarbodiimide; DIEA, N,N-diisopropylethylamine; DMF, dimethylformamide; DPA, diphenylamine; HOBt, N-hydroxybenzotriazole; MALDI-MS, Matrix-assisted laser desorption ionization mass spectrometry; MBHA resin, methylbenzhydrylamine resin; TFA, trifluoroacetic acid.
