# Supplementary material for: Design of Potent and Salt-Insensitive Antimicrobial Branched Peptides
Source: Polymers (Basel). 2023 Aug 29;15(17):3594. doi: 10.3390/polym15173594 (PMC10489980; doi:10.3390/polym15173594)
Supplement: Supplementary file 1 [file polymers-15-03594-s001.zip › polymers-2556065-supplementary.pdf]

**Supplementary information**

**Article title:** Design of Potent and Salt-insensitive Antimicrobial Branched Peptides

**Authors:** Janet To, Xiaohong Zhang and James P. Tam

Supplementary Table S1. Antimicrobial activity of dendrimeric D4R against ESKAPE bacterial pathogens under high-salt conditions.

| **ESKAPE bacteria** | **Species** | **Resistance gene** | **Remark** | **MIC (µM)** |
|---|---|---|---|---|
| ***E*nterococcus** | *E. faecium* DS01109 | MLVA 573322 ST117 | Vancomycin resistance | **1.03** |
| | *E. faecium* DS01463 | MLVA 573322 ST117 | Vancomycin resistance | **1.24** |
| | *E. faecium* DS01111 | MLVA 573323 ST17 | Vancomycin resistance | **0.81** |
| | *E. faecium* DS02415 | MLVA 573312ST203 | Vancomycin resistance | **0.9** |
| ***S*taphylococcus** | *S. aureus* EB 17917 | ST78 | Methicillin resistance | **0.52** |
| | *S. aureus* DM21713 | USA300 | Methicillin resistance | **1.35** |
| | *S. aureus* WBG04997 | - | Methicillin resistance | **1.51** |
| | *S. aureus* KKH 1-7942 | ST8 | Methicillin resistance | **0.86** |
| | *S. aureus* DM03076 | ST30 | Methicillin resistance | **1.61** |
| | *S. aureus* DR15686 | ST239 WITH ACME | Methicillin resistance | **1.04** |
| ***K*lebsiella** | *K. species* DU01301 | NDM, CTX-,-GP1 | | **0.47** |
| | *K. pneumonia* DU 07433 | NDM, CTX-,-GP1, DHA | | **0.31** |
| | *K. pneumonia* DR40294 | NDM, CTX-,-GP1 | | **0.12** |
| | *K. pneumonia* DB53879 | OXA-181, OXA-30, CMY | | **0.62** |
| ***A*cinetobacter** | *A. baumannii* M-96260-1 | CLONE II, OXA-23 | | **0.95** |
| | *A. baumannii* 6023688355 | CLONE II, OXA-23 | | **1.5** |
| | *A. baumannii* DM02370 | CLONE II, OXA-23 | | **0.36** |
| | A. baumannii DU35210 | CLONE II, OXA-23 | | **1.34** |
| | *A. baumannii* DU09676 | OXA-23 | | **0.6** |
| ***P*seudomonas** | *P. aeruginosa* DM14158 | ST964 IMP-1 | | **0.64** |

| | | | | |
|---|---|---|---|---|
| | *P. aeruginosa* DR26420 | ST654 IMP-26 | | **0.6** |
| | *P. aeruginosa* DM00727 | ST744 IMP-7 | | **1.3** |
| | *P. aeruginosa* DU37560 | ST233 IMP-1v | | **0.78** |
| ***E*nterobacter** | *E. coli* DS01878 | NDM-1, DHA | Carbapenem resistance | **0.54** |
| | *E. coli* DS00474 | NDM-1 | Carbapenem resistance | **0.75** |
| | *E. coli* DS00205 | NDM-1, CMY, DHA | Carbapenem resistance | **1.04** |
| | *E. coli* DS08293 | NDM-1, CMY | Carbapenem resistance | **0.63** |
| | *E. coli* DM13596 | CMY, CTX-M-GP1 | Carbapenem resistance | **1.33** |
| | *E. cloacae* DM15118 | NDM | Carbapenem resistance | **0.45** |
| | *E. cloacae* DM16303 | NDM, CTX-M-GP1 | Carbapenem resistance | **0.39** |
| | *E. cloacae* DM09800 | IMP | Carbapenem resistance | **1.0** |

Supplementary Table S2. Antimicrobial activity of dendrimeric and branched peptides with Orn- and Lys-based scaffolds under low- and high-salt conditions.

| | No. of strands | Salt Conc. | MIC (μM)* | | | | | | | | | |
|---|---|---|---|---|---|---|---|---|---|---|---|---|
| | | | Gram-negative bacteria | | | | Gram -positive bacteria | | | Fungi | | |
| | | | *E. coli* | *Pse.aeru-ginoa* | *Pr.vul-garis* | *K. oxy-toca* | *S.aure-Us* | *M. lu-teus* | *E. fae-calis* | *C. albi-cans* | *C.kefyr* | *C. tropi Calis* |
| α/ε-cascade D4R | 4 | Low | 0.6 | 0.5 | 1.0 | 0.4 | 0.8 | 0.5 | 0.8 | 0.8 | 0.9 | 0.7 |
| | | High | 0.7 | 1.2 | 1.9 | 0.9 | 0.6 | 0.7 | 1.8 | 0.8 | 1.3 | 0.8 |
| **Orn-scaffold** | | | | | | | | | | | | |
| O3R | 3 | Low | 0.9 | 1.9 | **6.9** | 1.7 | **9.6** | **5.4** | **49.8** | **6.4** | 0.8 | 2.1 |
| | | High | **8.7*** | **8.9** | **24.6** | 1.7 | **93.4** | **16.4** | **>500** | **18.2** | 1.4 | 1.9 |
| O4R | 4 | Low | 0.7 | 2.1 | **5.6** | 1.8 | **7.3** | 1.8 | **6.6** | 1.4 | 0.7 | 1.4 |
| | | High | **4.1** | **4.8** | 2.2 | 2.4 | 2.0 | **6.9** | **>500** | 1.6 | 0.8 | 1.5 |
| O5R | 5 | Low | 0.9 | 2.0 | **8.1** | 1.8 | **5.4** | 1.3 | **10.2** | 0.9 | 0.7 | 1.1 |
| | | High | 2.3 | **3.1** | **19.2** | 2.4 | **6.5** | 1.7 | **>500** | 1.5 | 0.7 | 0.7 |
| **Lys-scaffold** | | | | | | | | | | | | |
| K3R | 3 | Low | 0.7 | 0.9 | 0.6 | 0.7 | 0.6 | 0.7 | 1.0 | 1.0 | 0.8 | 1.1 |
| | | High | 0.8 | 0.7 | 1.0 | 0.8 | 0.6 | 0.8 | 1.3 | 0.7 | 0.7 | 0.6 |
| K4R | 4 | Low | 0.7 | 0.8 | 1.1 | 1.3 | 0.8 | 0.6 | 0.5 | 0.7 | 0.9 | 1.5 |
| | | High | 0.7 | 0.9 | 1.0 | 1.2 | 0.9 | 1.0 | 1.5 | 0.8 | 0.9 | 1.2 |
| K5R | 5 | Low | 0.7 | 0.8 | 1.2 | 0.9 | 0.8 | 0.6 | 1.3 | 0.7 | 0.9 | 1.1 |
| | | High | 0.6 | 0.8 | 0.7 | 1.3 | 0.7 | 0.8 | 1.4 | 0.8 | 0.9 | 1.3 |
| **Iso-Orn scaffold** | | | | | | | | | | | | |
| iO3R | 3 | low | 1.3* | **5.8** | **8.3** | 2.2 | **17.2** | **3.1** | **51.6** | **4.8** | 1.3 | 2.8 |
| | | high | **12.8** | **3.2** | **27.6** | **5.4** | **56.2** | **8.8** | **>500** | **12.4** | 2.0 | 2.3 |
| iO4R | 4 | low | 0.9 | **4.4** | **4.7** | 1.6 | **6.7** | 2.1 | **5.4** | 1.3 | 0.8 | 1.4 |
| | | high | **4.4** | **4.0** | **23.2** | **6.8** | **4.8** | 2.0 | **>500** | 1.4 | 0.8 | 0.8 |
| iO5R | 5 | low | 0.7 | 1.0 | **8.2** | 1.2 | 1.9 | 0.8 | 2.2 | 0.6 | 0.6 | 0.9 |
| | | high | **9.2** | **3.8** | **7.8** | **6.2** | **6.4** | 2.0 | **>500** | 0.8 | 0.5 | 0.6 |
| **Iso-Lys scaffold** | | | | | | | | | | | | |
| iK3R | 3 | low | 0.6 | 0.7 | 0.7 | 0.7 | 0.5 | 0.6 | 0.5 | 0.7 | 0.8 | 0.8 |
| | | high | 0.4 | 0.8 | 0.5 | 0.7 | 0.7 | 0.7 | 0.8 | 0.8 | 0.7 | 1.2 |
| iK4R | 4 | low | 0.4 | 0.5 | 0.8 | 0.5 | 0.7 | 0.9 | 0.8 | 0.8 | 0.8 | 0.5 |
| | | high | 0.4 | 0.7 | 0.7 | 0.7 | 0.6 | 0.8 | 1.4 | 0.9 | 0.8 | 1.3 |
| iK5R | 5 | low | 0.5 | 0.6 | 0.6 | 0.5 | 0.8 | 1.0 | 0.8 | 0.8 | 0.6 | 1.2 |
| | | high | 0.5 | 0.8 | 0.7 | 1.3 | 0.7 | 0.6 | 1.6 | 0.8 | 0.7 | 0.9 |

* When MIC is >3 μM, the number is shown in bold.
